# Extra-Limites
*The Extra-Limites of this Number we give at our own expence, as a bonus to Subscribers, and a mark of respect to the authors. We shall be ready to honour select Essays of merit with this distinction, from time to time, in the succeeding Numbers of our Work, and beyond the established limits.—*Editors.*


**Published:** 1825-04-01

**Authors:** 


### EXTRA-LIMITES.*

### I.

*Observations on the Dangers liable to result from the* less frequent *Use of the Trephine, in Fractures of the Cranium with* Depression, *illustrated by Cases.* By Richard Wise, Member of the Royal College of Surgeons in London, Extraordinary Member, and late President of the Royal Physical Society in Edinburgh.

Dico quòd per amotionem ossis non additur malum malo ; sed removetur malum a malo ; nec etiam in osse operari est tam grande malum, ut putant aliqui.

*Berengarius,* cap. xxv.

The dangers which so often succeed to wounds of the head, and the frequent obscurity of their commencement and progress, have, in every age, from the time of Hippocrates, to the present day, been universally acknowledged : the mode of treatment, however, has, unhappily, ever been, more or less, a subject of controversy, and at no period, perhaps, has this been more conspicuous, than about the close of the last century.

The propriety of the general practice of applying the trepan in fractures of the cranium, which, about that time, seemed to have been pretty well established, not only in this country, but throughout the continent of Europe, was at once disputed and put down by Desault, who even went so far as almost to proscribe its use in every case. This innovation, from one at the head of his profession in the metropolis of France, both as a practical surgeon and a lecturer, was not only readily and implicitly received by his numerous pupils at home, but was also soon favourably noticed, and partially supported, in the lectures and writings of some of the most eminent teachers of surgery in this kingdom, who now supposed it probable that the operation had been performed too frequently and unnecessarily by Mr. Pott and others.

But whether Mr. Abernethy, in his "Surgical Observations on Injuries of the Head," intends to discountenance the use of the trephine in fractures of the cranium *with depression,* so far as Mr. Samuel Cooper

* The Extra-Limites of this Number we give at our own expence, as a bonus to Subscribers, and a mark of respect to the authors. We shall be ready to honour select Essays of merit with this distinction, from time to time, in the succeeding Numbers of our Work, and beyond the established limits.—*Editors.*

appears to have conceived, seems to me questionable. For the deductions which Mr. Abernethy has drawn from such of his cases as did well, without the operation, should, I think, be received rather as speculative suggestions, held out for the consideration of others, than as established practical precepts, of the value of which that gentleman was himself, fully satisfied.

The injunctions, indeed, laid down by Mr. Cooper, are the most unequivocal, and decided. *" That every surgeon cannot be too fully impressed with the following truth, that *existing symptoms* of *dangerous pressure on the brain can alone form a true reason for perforating the cranium.*" † " Before beginning the description of the operation, I think it highly proper to remind the reader of what has been so forcibly dwelt on in the" article " Head, Injuries of." " That the removal of the pressure off the brain, which pressure must also actually occasion dangerous symptoms, can form the only true and vindicable reason for employing the trephine, or sawing away any portion of the skull."

" It should never be undertaken but in the most pressing circumstances, and when the symptoms unequivocally show that a dangerous degree of pressure on the brain exists." " The symptoms indicating compression of the brain, and these symptoms must also be urgent and alarming, can alone justify an examination of the fracture."

With the greatest deference to the judgment, experience, and elaborate research of this respectable, and popular writer on surgery, I would beg to ask, whether, in the above practical inculcations, the attention is not *too exclusively directed* to the *immediate* symptoms of *functional* derangement of the brain, as *alone* affording a true reason for perforating the cranium, or as justifying even the examination of a fracture, as if this were the only circumstance, in such cases, from which danger could be apprehended: thereby, in depressed fractures, unaccompanied by such immediate symptoms of compression, setting wholly aside, and losing sight of the inflammation, and often fatal consequences that may result from allowing the irritating pressure of broken bone to remain on the brain and its membranes unelevated.

Could Mr. Cooper satisfactorily prove, that the slightest degree of pressure, from depressed bone, would at all times give rise to immediate functional derangement, then, indeed, might we, inasmuch as it would also inform us of the pressure, which might *induce inflammation,* with safety, perhaps, rely on this indication, for the performance of the operation. But no, this gentleman, himself, tells us, that it is extraordinary and unaccountable, but no less true, that no calculation of the functional derangement can be made by the degree in which a part of the skull is depressed, and, in confirmation of this, quotes a late eminent writer,‡ who has detailed some most interesting cases, as occurring amongst the wounded in the battle of Waterloo, wherein very *considerable depressions*

---

* Surgical Dictionary, 3d Edition, Article " Head," page 897.

† Article " Trephine." ‡ Dr. Thomson.

produced no *stupor*, *paralysis*, or *loss* of *memory*,—in short, no *functional derangement.*

Yes; such are the physiological and pathological peculiarities of these parts, that it is possible for a portion of broken bone to be so depressed, as not only to bear considerably on the dura mater, but to pierce that membrane, and even to enter the brain itself, without producing immediate functional disturbance, or any other symptom to indicate the true nature, or extent of such an injury. Nay, it frequently happens, that the irritating pressure of the bone thus situated, gives rise to inflammation, and suppuration, terminating, in death itself, without causing pain, or exhibiting any other sign, to warn us of the danger, till the patient is beyond the reach of help.

This is indeed, an assertion, in the belief of which, we cannot, at first, perhaps, but feel somewhat sceptical, particularly when we think of the sufferings, which accompany even the slightest inflammation of the eye—of the ear—of the finger, in paronychia—or of any external part of the body.—But when we call to our recollection how abundantly all these parts are supplied with nerves, whilst the membranes of the brain have, in all probability *none*, it cannot but, in some measure, reconcile us to these discrepancies.

Numerous have been the experiments performed by Haller, Zinn, and other eminent physiologists, in proof, not only of the insensibility of the membranes, but also of the brain itself.* "The dura mater has, by way of experiment, been pinched, and injured by every possible contrivance, both by mechanical and chemical stimulants, yet the animals have given no signs of pain." Haller says "Dura mater absque sensu, aut irritabilitate, et absque *nervis* est."†

*Case* 1. In proof of the *pathological obscurity*, Mackreen gives a remarkable case, in which several fragments of the bone penetrated even to the ventricles of the brain, yet the man walked home unassisted. He lived from the 23d of February, to the 11th of March, during which time, there was daily lost a spoonful of the very substance of the brain—his speech—intellect—and judgment being all along perfect—nor was it *till death* was fast approaching, that the delirium—fever—or other symptoms came on.

*Case* 2. In the "Medical Repository" for October 1824, Mr. Dickson relates the sudden death of a sailor, aged 50, in the act of rowing. On the removal of the dura mater, falx, &c. after death, there was found upon the middle lobes of the brain, where they came in contact with the falx, a considerable quantity of thick, grey coloured pus, most of which lay in a cavity formed by ulceration of the approximating portions of the middle lobes. That portion of the falx, which bisected the abscess, was diseased, and was pervious to the abscess.—The convolutions

* Bell's Anatomy, Vol. 1. † Primæ Lineæ Physiologiæ, Cap. x. cccxx.

of the brain abounded in serum, and the lateral ventricles contained each an ounce and a half of serous fluid. Yet, from the man's messmates, it appeared that he had not complained for *some months* previous—that he had done his duty—eaten his allowance—and drank his "pint" like others.

In the "Medical and Physical Journal" for January 1821, there is a case given, wherein the operation of trephining was performed about a *fortnight* after the receipt of the external violence; and it was stated in evidence, at the coroner's inquest, "that the patient followed his accustomed bodily labour, for five days after the infliction of the injury—but, that on opening the body after death, the skull was found to be *extensively fractured*—and that several spiculæ of bone had actually *been driven into*, and produced *disorganization of the substance of the brain* itself, although *fourteen days* had elapsed before the usual symptoms manifested themselves."

Though the brain and its membranes may thus sustain the severest local injury from depressed bones, without giving rise to *immediate pain*, or *functional disturbance*, are we to infer, that the continuance of pressure, because at first not openly offensive, will not excite inflammation? —Would a fragment of glass, found thrust into a *palsied* limb, be deemed innoxious, merely because the patient had *no pain*, to complain of?

Were it proved, or even contended, that the brain and its membranes are equally unsusceptible of inflammation as they are of immediate pain, from *irritating* pressure, we might, indeed, be less anxious to enquire into the precise nature of depressed fractures. *" But, of all parts of the body, these are most prone to inflammation, especially where the irritation is made *through the bones*, though, in no part is its progress, and that of suppuration, so obscure, so insidious, or so deceptive and, when far advanced, so unmanageable, and, where it so often baffles our art." Indeed, every hour convinces us, that inflammation may be induced even by the slightest mechanical pressure, and, we should frequently suffer from its operation, but for the ever protecting sensibility, with which every external part is endued. Yet, however indispensable to our immediate security this general diffusion of nerves, such a supply to the membranes of the brain, amply protected as they are, by the bony walls of the cranium, would seem unnecessary, though, were they equally so, their pathology, would, no doubt, be less obscure. Nor should we then find so many unhappy cases on record, where the effects of injuries of the head, had proceeded to an irremediable extent, without one symptom to denote, or warn us of the existence of danger.

In further proof that depressed fractures, though considerable, do not always give rise to immediate pain or functional disturbance,—of the dangers that may result therefrom, notwithstanding—and of the insidious progress of inflammation, consecutive thereto, I will relate, briefly, the following cases.

*Case* 3. Hercules Bishop, aged 14, in descending a copper mine, by

---

* Bell's Operative Surgery.

a ladder, received a small wound in the head, by the fall of a stone. He took little notice of the accident, and continued to his labour, without complaint, for the next *twelve days*, when he became ill. My father was requested to visit him, for, what his friends thought, a fever, as they had not the least idea, that the wound of the head, which had not even been seen, or dressed, for some days, was at all connected with his present illness; for it is probable the circumstance of his having received it, would not have been noticed, had not the questions put, brought it to their recollection. His pulse was quick, and skin hot. He had low muttering delirium, with stupor, and occasional twitchings. On examining his head, there was found, over the middle of the left parietal bone, a small wound, the edges of which, were slightly inverted, and flabby. On introducing the probe, there was discovered a fracture with depression. The integuments were now laid back, and the fracture exposed, when a portion of bone, about half an inch in length, and a quarter in breadth, was found, not only depressed, but partly sunk into the dura mater. The trephine having been applied, and the depressed portion of bone elevated, a considerable quantity of fetid matter was discharged, and the dura mater was observed to have a gangrenous appearance. The operation did not relieve any of the symptoms. He lingered till the following morning, when he expired.

*Case* 4. "An herb-man was struck upon the forehead, near the coronal suture, by a blunt instrument. Subsequent to the blow, he experienced a tendency to syncope. He came into the hospital, and, till the *eleventh day*, there were no untoward symptoms, but every thing portended a favourable issue. On that day, however, he was seized with violent fever, which commenced with rigor, and excessive vomiting of bile; and he had a daily recurrence of febrile symptoms till the fourteenth day from the blow, when some hebitude was observable. A few hours afterwards, he became senseless, respired with difficulty, and died before the end of the day.

*Dissection.* The os frontis was slightly depressed, and an unequal and sharp lamina had been detached from its inner surface, and had wounded the dura mater, and, consequently, pus had formed betwixt that membrane and the pia mater, whence it had descended from the vertex nearly to the base of the cerebrum, and also to the bottom of the cerebellum, on the corresponding side.*"

*Case* 5. William Holman, a child aged two years, fell through a hole in the floor, into a room underneath, on its head, a slight tumour soon arose, and vomiting came on. The child lay in a comatose state several hours, after which he gradually became sensible, and apparently as cheerful, and as free from complaint, as he had ever been. Things remained in this state more than a fortnight, when he became fretful, restless, and feverish. The tumour, which was small, for some time

* Cooke's Morgagni, vol. I. p. 216.

after the accident, had, during the last two or three days, become larger, and was now the size of an egg, and soft as if containing pus. In this state, the child was brought to me. Having had the history of the case from the mother, my partner, Mr. Vincent, and myself examined the tumour, and conceiving it to be an abscess, I laid it open, when, to our mutual surprize, a great quantity of brain, and fetid matter was discharged. Having introduced a probe, we readily discovered a fracture, with depression, and, on exposing it, found the sunken portion of bone had penetrated the membranes into the brain. As it was loose, we removed it with the forceps, and more brain was discharged. The child did not appear to be relieved by this, but, on the next day, seemed in a dying state; however, in this melancholy condition, he passed the four following days, and then expired. From the period of opening the tumour, to the death of the child, brain mixed with blood, was continually discharged, and so offensive was the stench proceeding therefrom, as to render it almost impossible for any one to enter the room where the child lay, the fond mother only excepted, who continued to her infant the most unremitted attention to the last moment of its existence.

*Case* 6. (The 11th of Mr. Abernethy). The coachman. A piece of bone, in this man, the size of a sixpence, was slightly depressed. No bad consequences appeared for two months, when the bone was removed. The patient, after lingering some time, expired. The whole right hemisphere of the brain was found reduced into a pulpy and fetid mass.*

*Case* 7. (I was favoured with this, by Robert Liston, Esq. lecturer on surgery). "A coachman in Edinburgh was knocked down off his legs. He fell on the corner of a stone, which broke off, and stuck into the bone. The stone was picked out—he was bled, and the wound dressed. He continued to drive his coach till the *following Sunday, without complaint,* when he returned home shivering. I (Mr. Liston) was now sent for. On examining the wound, which was situated about the posterior superior angle of the right parietal bone, a portion of which, about an inch long and half an inch wide, was found depressed half an inch. The integuments had separated, and were pulpy. The bone was denuded and pale. The pupils dilated, pulse 60, and oppressed. He was bled ad uncias xij.—blood buffy—somewhat better, though there is stertor. *Monday,* I saw him with Dr. S. and Mr. R. —Returned at 8, p. m.—applied the trephine, and raised a number of splinters from the dura mater, chiefly from the inner table. The membrane was found depressed, and the splinters mixed with curdy matter —The dura mater rose to its level. He is now more sensible.—*Tuesday.* Answers questions unwillingly, and complains of head-ache—pulse quick and full—pupil contracted, and more sensible to light—

* I must beg to refer the reader, for a full detail of this and the other subsequent cases, already in print, to the works in which they are respectively given.

restless during the night—no stool. Emittatur e brachio sanguis ad uncias xxiv.—a large spoonful of a saline opening mixture, omni horâ. *Wednesday.* Seized with convulsions last night, relieved by bleeding, but they returned, and he died at one this morning. Sectio.—Dura mater sound—about *four ounces of flaky matter* lying over the surface of the tunica arachnoides, brain sound."

The above are not only valuable, as well marked-cases of fracture of the cranium with depression, not giving rise to immediate functional disturbance, and of the obscure progress of inflammation; but they must also impress us forcibly, that the inflammation and its fatal effects arose chiefly from the *irritating pressure* of *unelevated broken bone*, and, on comparing this with the seven cases presently to be detailed, may we not very fairly infer that the unhappy consequences might have been *prevented* by an early application of the trephine?

*Case* 8. John Edwards, aged 23, whilst in a scuffle, had a stone thrown at him, by a person unknown. It struck him on the hinder part of the head, just below the lambdoidal suture. He was stunned immediately after the accident, but soon recovered, and was able to walk to my house, the distance of half a mile. On examining the wound with a probe, I at once discovered a fracture with depression, and, by exposing the injury, a small triangular portion of the occipital bone depressed about a quarter of an inch. I applied the trephine, elevated the depressed portion, and brought the integuments together, which readily united by the first intention. No exfoliation took place. In the space of three weeks, he was able to return to his labour.

*Case* 9. Henry Skewes, aged 20, was struck on the head with the point of a reaping hook. On examining the wound, which was about the posterior superior part of the parietal bone, near the longitudinal sinus, I found a fracture with a small portion of bone depressed. There was no functional disturbance. On attempting to elevate the depressed part, after the application of the trephine, it broke off. The edges of the wound were brought together, and it healed in about ten days, without a bad symptom supervening.

*Case* 10. (The 10th of Mr. Abernethy). The drunken woman. A circular piece of bone was beaten in, to the depth of a quarter of an inch. The trephine was applied—the shattered and depressed bone removed, as was also a clot of coagulated blood, as large as a walnut. She shortly became perfectly well.

The four remaining of these cases are, besides, particularly interesting, and deserving attention, inasmuch as they must convince the most timorous that extensive exposures of the dura mater, or wounds of that membrane, or even of the brain itself, from depressed bone, are not necessarily fatal—or so frequently dangerous, or, indeed, so liable to give rise to alarming symptoms, as they are, by some, supposed and represented, provided the same *preventive* measures be, in all such wounds promptly attended to, which are ever judged indispensably necessary, in those of every other part of the body—namely, the careful removal, in the first instance, of all *foreign bodies*, of whatever nature, which might prove a *source* of *irritation*.

*Case* 11. Richard Kneebone, aged 14, got his head crushed in a thrashing machine. I found the scalp reverted from the left ear over the right side of the head, exposing nearly the whole of the parietal bone, the anterior superior part of which, about three inches over, was deeply depressed. He lay quite insensible, his pulse slow, and the pupils dilated, indeed, he appeared altogether so much like one in a dying state, that the friends, at first, objected to my doing any thing for him. I found it necessary to make two perforations, to elevate the sunken portion which broke off, in the act of raising it. The dura mater, now so *extensively exposed*, rose to its level, and the boy soon became somewhat more sensible. The scalp was brought down, as nearly as possible, to its former situation, and was secured by a few stitches, except at the inferior angle, which was much contused. Pretty much blood was lost from the accident, and during the operation. The wound being dressed, a purgative was ordered. He slept the whole of the night, and, by the next morning, his senses had completely returned. On removing the dressings, the third day, a union of the edges had taken place, except at the lower part. The boy, notwithstanding this extensive injury, had not a single bad symptom after the operation, and was, in about a month, quite recovered.

*Case* 12. A soldier, of the 44th regiment, who was wounded by a musket ball, at the battle of Waterloo—a case of Mr. Samuel Cooper.* "The splintered bone was, at one point, driven more than half an inch into the membranes and substance of the brain. The trephine was applied, and on the same day this patient got up, and dressed himself, without leave of the medical officers, and never had a bad symptom afterwards."

*Case* 13. A man, in descending a copper mine, was struck on the head by the fall of a stone. The force was so great as not only to fracture the bone, but to force one end of the portion through the membranes, into the substance of the brain. The application of the trephine was found necessary for the removal of the sunken portion, which being effected, some brain was discharged through the opening, as there was also, during several of the first dressings, yet the patient, to the great surprize of my father, so far recovered, in a few months, as to return to his labour.

*Case* 14. (Mr. Crawford's.*) In this boy, about three years of age,

* Surgical Dictionary, article "Trephine."
* Edinburgh Medical and Surgical Journal for January, 1819.

the wound was *extremely small.* There was, however, an evident depression—though *no evident symptoms of compression.* Notwithstanding the generally received rule, says Mr. Crawford, *not to interfere, unless such symptoms do actually occur*, we did not hesitate to make the necessary incisions, for examining the state of the skull. More than two inches in length, and one in breadth, of the left parietal bone was found comminuted, and sunk near an inch into the brain. The cavity formed by the deepest part of the fracture, was filled with protruded brain, the bulk of a walnut, consisting both of the cortical and medullary substance. The trephine was applied, the depressed portion elevated, and the rugged edges of the surrounding bone taken away, the scalp was brought together, and, though there was hernia cerebri, the boy perfectly recovered.

It may be said, had a strict antiphlogistic treatment been pursued in some of the unhappy cases given previously to these just detailed from the moment the injuries were received, the inflammation might have been so moderated, if not altogether prevented, as to have obviated their fatal effects, and, in support of this, the cases of fracture with depression, from Mr. Abernethy, Mr. Latta, and others, which eventually did well without the use of the trephine, may be brought forward—yes; were the use of the trephine, from this day, to be wholly proscribed, and, in future, all cases of depressed fracture, from the slightest to the very worst possible, to be submitted to the antiphlogistic treatment *only*, no doubt, there would in a few years, be many extraordinary cases of recovery to relate, in addition to those already recorded, in support of the practice. But such apparently miraculous recoveries, may, I think, with considerable probability, be accounted for, from the favourable operation of adventitious circumstances. There may, for instance, be fractures with the most palpable depression, where the fractured portions may be so readily susceptible of the constant pulsatory motion of the contents of the cranium, as to be thereby speedily raised to their level, without inducing inflammation.

Two cases are mentioned by Dr. Munro, in his lectures, as communicated to him by a respectable surgeon residing in the vicinity of Edinburgh. The depressions were very considerable, and, in each, the gentleman urged the immediate necessity of applying the trephine. This, however, the friends refused to comply with. The patients, notwithstanding, went on well, and the depressed portions *were soon restored to their proper level.* Or further, though a depressed bone may be unyielding to the elevating force just alluded to, it may possibly present a somewhat convex, or less rugged, and comparatively inoffensive edge, to the dura mater. The patient, at the same time, may be of a phlegmatic habit, and, consequently, less disposed to inflammation, and this also, happening in a young subject, the brain, during a strict antiphlogistic treatment, might readily and safely adapt itself to the depression.

That a general reliance, however, on such circumstances, precarious as they must necessarily be, would frequently expose patients, not only to much danger, but to irretrievable mischief, must be obvious. Saying nothing of the chronic affections of the brain, as epilepsy, &c. which such unelevated depressions may probably give rise to.*

The two following cases, though they eventually did well, without the operation, were, at the time, however, seemingly exposed to much danger.

*Case* 14. (Mr. Latta's.) A female, of six years, fell from a table; the wound in the scalp, on the left side, over the frontal bone, was pretty large, with an evident depression, for more than half an inch in diameter. The child lay comatose for some hours, and vomited several times. By the next day, she seemed tolerably recovered, and went on well till the *sixteenth*, when she was seized with a chilliness and shivering, which, in a short time, was succeeded by heat and restlessness, attended by a quick and hard pulse. She, however, under a strict antiphlogistic treatment, recovered, and, in a month, the *depressed bone was completely elevated*.†

*Case* 15. (7th of Mr. Abernethy.) A lad, 18 years of age. In this case, it was impossible to apply the trephine below the fracture, which was situated about a quarter of an inch above the zygoma. The bone was depressed, about the eighth part of an inch. There was no functional disturbance. He went on well till the evening of the second day, when he had become delirious, and could scarcely be kept in bed. His skin was hot, the pulse frequent and strong; he was, therefore, immediately and largely bled. He now became quiet and manageable, but, by the next morning, his answers were incoherent, pulse frequent, his skin hot, and his tongue dry. The bleeding and purging were repeated, and, at night, a blister was applied to his neck. From this time, he gradually became well.

If such cases had been given to show that fractures of the cranium, even with considerable depression, may, in some of the more favourable instances, by vigilant attention and active treatment, do well, without the use of the trephine, where, from the fracture extending into the base of the cranium, or otherwise, the operation might be rendered unsafe or impracticable, they might, no doubt, prove highly useful, by serving to teach us not to despair under such circumstances. But, if they are recorded, whether to establish the inoffensiveness of pressure on these parts—the sufficiency of the resources which the brain has within itself, of removing such irritating pressure, by raising the depressed bone to its level—to set forth the powers of the lancet, and its auxiliaries—to show that the solicitude which Mr. Pott evinces is unnecessary—or, whatever they may be intended to establish or illustrate, with the view of discountenancing the use of the trephine; let us first reflect on the general nature of the depressed fracture—how often the portion of bone

---

* Vide Dr. Abercrombie's paper on Chronic Inflammation of the Brain, —Edin. Med. & Surg. Journal, 1818.

† Latta's Surgery.

is so impacted as to require considerable force, even by the elevator, to raise it to its level—how utterly inadequate, in such cases, the pulsatory force of the contents of the cranium, to restore the bone to its situation—and, further, how insufficient the lancet, and all the adjutory means with which it may be accompanied, and to whatever length enforced, to prevent the accession, or wholly to arrest the progress of inflammation, in such injuries—I say, let us call to mind these important circumstances, before we implicitly adopt a practice, however highly authorized, which, in many cases, must be fatal, and in all, most hazardous.

The operation of the trephine, when skilfully and cautiously performed, and not under circumstances decidedly unfavourable to its success, as it would undoubtedly be, when had recourse to, as a dernier resort in chronic diseases of the brain, or in crowded hospitals, notorious for the malignity of the air, as was the Hotel Dieu*, for instance, in the time of Desault; has never, that I am aware of, been fairly and positively proved of that dangerous nature, as to warrant the *hazardous practice of leaving a palpably depressed bone unelevated.* Were it so, surely we should hardly expect to find in almost every age, practical surgeons, eminent as well for their long experience, skill, and respectability, as for the public situation which they held, deliberately advising the application of the trepan again and again on the same patient. " If, by making one orifice," says Mr. Sharp, " you cannot raise all the depressed parts, you must make a second, and a third, and continue doing so, till you have reduced the whole cranium even. There is frequently occasion to repeat it twice or thrice, and it has been done twelve times, nay oftener, with success, which," continues Mr. Sharp, " I mention to shew the little danger there is, either in sawing the skull, or in exposing the dura mater and brain, when the *pressure* is taken off."†

I practised in a mining district, in the county of Cornwall, for many years, where the labourers, from the nature of their employment, are particularly exposed to injuries of the head. The number of fractures of the cranium which consequently fall to surgeons there, is not, perhaps, exceeded, if equalled, any where in the kingdom. My father, for a series of years, had the care of a great number of these miners, and a gentleman, in the same town with myself, was still more extensively engaged. They are both practitioners of long and tried experience. They trephined in all cases of depressed fractures, and their practice has always been decidedly successful.

And further, if Mr. Pott, who, of all others, perhaps, employed the trepan the most frequently, did not think the operation " painful or ha-

---

* " L'Hotel Dieu no longer deserves to be called a " Store House of Corruption and Disease. A revolution has taken place in the internal administration, as well as in the Surgical Practice of this institution."—*Medical Schools of Paris, by J. Cross, M. R. S. L &c. Vide also Observations on English and French Surgery, by R. J. Roux, page* 51.

‡ Sharp's Surgery, page 144.

zardous," and that too, at a time when it was the practice wholly to remove a portion of the scalp previous to its performance, surely we, in the present day, on availing ourselves of the now well-established practice of preserving the scalp, must think it less so.

It is scarcely necessary to observe here, that Mr. Pott trepanned in every case of depressed fracture—in fissures—and severe contusions of the skull—and this, not only when urgent symptoms of functional disturbance demanded an immediate performance of the operation, but also when no such indications accompanied the injury, solely with the view to *prevent* this consecutive inflammation—effusion—suppuration—caries—and exfoliation of the bone—and separation of the dura mater, with all their mischievous consequences, which he, from very long experience, knew was so liable to succeed to the irritating pressure of broken bone, &c. when suffered to remain unelevated.

" No doubt," says Mr. Pott, "that although by establishing it as a general rule to perforate in all cases, some few would now and then be subject to the operation, who might have done well without it; yet, by the same practice, many a valuable life would be preserved, which must have been lost without it, there being no degree of comparison of the good to be derived from it, when used early as a *preventive*, and what may be expected, if it be deferred till an inflammation, and symptomatic fever make it necessary."

Directly in opposition to this *preventive* practice of Mr. Pott, I am aware the highly influential authority of Mr. Abernethy too, may be quoted, who says,—" Whenever the patient *retains his senses perfectly*, I should think it improper to trephine him, unless *symptoms arose* that indicated the necessity of it"—but let the young and inexperienced surgeon, nevertheless, beware how he passes by the *timely use* of the trephine, in placing an undue reliance on its efficacy when performed on the actual appearance of secondary symptoms of compression. For, in the generality of such cases, it will be found that these symptoms arise from the pressure of matter on the brain, and " this state of things," to use the words of Mr. Samuel Cooper, "cannot come on till after the inflammation of the brain and its membranes has prevailed some time."* It is true, the operation, by giving vent to the confined matter, will sometimes afford temporary relief, and where the previous inflammation may not have been extensive, the patient may also eventually recover, as has been exemplified in the fortunate cases of Mr. O'Halloran. But it must not be concealed that, however justifiable and proper the use of the trephine, where the surgeon is not called till this unhappy period, that by far—far the greater number of such cases terminate fatally.

What permanent advantage can, indeed, be rationally expected from the operation, at a time when the brain is in a great measure destroyed ?

The following very interesting case, from the publication of Mr. Cross, already referred to, is so much in point, that I hope my transscribing it will require no apology.

---

* Surgical Dictionary, page 923, 3rd Edition.

*Case* 16. "A woman was admitted at L'Hospice de Perfectionment, on the twentieth of January, with fracture and depression of the frontal bone. She had scarcely recovered from the concussion attending the injury, but was sensible, and not labouring under any symptoms of compression. Dubois applied the trepan, and raised the depressed portion to its proper level. The wound was dressed daily, with lint and common cerate. On the evening of the 6th of February, this woman, who seemed to have gone on very well since the operation, was attacked with vomiting, followed by fever, and low delirium. She died on the morning of the ninth." Taking this case thus far, certainly it cannot be said to be favourable to the use of the trephine, for the patient died, though the surgeon had recourse to it immediately after the accident—but mark the sequel. "Forty hours after death," continues Mr. Cross, "the head was inspected at the public theatre, before the students left the hospital. The fracture of the frontal bone had extended into the orbit—a spicula of bone had pierced the dura mater, and was still remaining "enfoncée dans le cerveau."* There was pus between the hemispheres of the brain, and effused from the injured parts, as far back as the cerebellum, and a portion of the right anterior lobe of the cerebrum, had a gangrenous appearance."

What opinion will the unprejudiced reader now give respecting the cause of the death of this woman? Will he, for a moment, hesitate to lay it to the unelevated portion of broken bone, discovered piercing through the dura mater into tbe brain itself? Yet the patient continued until the evening of the sixth of February, *seventeen days after the accident*, apparently not only free from danger, but from any unpleasant symptom whatever, when she was suddenly seized with vomiting, followed by fever, and, on the morning of the 9th, little more than two days after the attack, expired. Now, permit me to ask, when could the inflammation, which ended in this extensive diffusion of matter, and gangrenous state of the cerebrum, have commenced? I presume, no one will say on the evening of the 6th of February, the time when she was attacked with vomiting. For what analogy have we, that can warrant such an inference? If, then, we give up this point, its commencement must be dated farther back, and that to a period when the patient had no pain, fever, or other symptom to complain of, and was thought in a fair way of recovery. That the inflammation, which ended in so wide a diffusion of pus must, notwithstanding the absence of symptoms, have begun soon after the infliction of the injury, can hardly be questioned. To think otherwise, must be in direct opposition to the plainest facts in surgery. Have, we, for instance, on the one hand, ever seen, heard, or read of the inflammatory, suppurative, and gangrenous stages, *all* running their course in the brain in the short space of two days, from such a cause? Or, if so, on the other, can it be supposed that inflammation was not likely to commence during the first seventeen days after the accident, under the circumstances disclosed in the dissection, when

* Sunk deep into the Brain.

a piece of broken bone was found depressed, actually piercing the dura mater, and sunk deep into the brain, and this too rendered doubly noxious by the constant pulsatory motion of the contents of the cranium against the fractured and unyielding portion? Impossible. Did I want another case further to corroborate those already adduced, to prove that the brain and its membranes may not only be wounded by depressed fragment of bone, but that this fragment may actually remain piercing the parts, without occasioning immediate symptoms of compression—and further, that this shall, by its irritation, give rise to inflammation, suppuration, and gangrene itself, without pain, fever, or other symptoms by which we might apprehend the presence of such an evil, or the commencement or progress of its fatal consequences, surely this of Mr. Cross has supplied me.

The surgeon prejudiced against the operation, had it not been for the *examination after death*, would here have had another case to relate, in proof not only, perhaps, of the inefficacy, but of the positive injury of the trepan.

Is there not considerable probability that many of the fatal cases* recorded, may have been of a similar nature?

It may probably be said that I have insisted too much, throughout this paper, on the pathological obscurity of the brain and its membranes. If I have said much, it is because the non-trephiner has said far—very far too little, on this important practical point. That such pathological obscurity, however, is not observed in all cases, I most readily acknowledge—that the commencement and progress of inflammation of the brain and its membranes are sometimes apparently indicated by a set of corresponding symptoms, all have agreed; and by no one have these symptoms, perhaps, been more accurately given, than by Mr. Pott himself—but, that the certainty of their appearance cannot be relied on, to guide our early treatment, the case of Mr. Cross, just detailed, and the cases Nos. 3, 4, 5, 6, and 7 in this paper, together with the opinions of many of the most experienced surgeons, unquestionably prove.

But, let us suppose that the commencement and progress of inflammation were in all cases clearly and uniformly indicated. Would this enable us to meet the exigency in every case?—By no means. It might, indeed, in some instances, obviate the no inconsiderable inconveniences of exposing the patient to the full use of the lancet, and the whole train of its auxiliaries, unnecessarily—but it could never prevent the putting in practice such depleting measures, in others, unsuccessfully. For no one would presume he could prevent, however highly he may rate the powers of the lancet, inflammation taking place in a part on which an unyielding depressed fragment of bone presses with its rugged edge, saying nothing of such cases in which the depressed fragment remains actually piercing the parts.

From the view, then, which I have taken, throughout this paper, of the physiological and pathological peculiarities of the brain and its

---

* See Case.

membranes, the mode of practice I should adopt, will readily be anticipated. With a few exceptions only, and these under peculiar circumstances, especially in young subjects,* I should apply the trephine in every case of fracture of the cranium, with palpable depression, particularly beyond the walls of an hospital, whether *accompanied with symptoms of compression or not.* When accompanied with symptoms of compression, I should perform the operation with *two* intentions; first, to free the brain from the pressure giving rise to such apoplectic symptoms, by its acting more or less on the whole of that organ; and, secondly, to remove the *irritating pressure* from the parts on which the depressed bone *actually bears*, for the same obvious reason, that I would remove a foreign body from a wound in any other part of the body.

In cases unaccompanied by such *functional derangement*, ever bearing in mind, that the most *dangerous irritating pressure may exist, notwithstanding the absence of such symptoms*, I should also apply the trephine, with the second intention, namely, as a *preventive*.

I feel thus desirous to direct the attention to the *irritating* pressure which must so frequently take place in depressed fractures, and its often pernicious consequences, when suffered to remain unelevated, since it appears to me to have been wholly *overlooked*, in the practical inculcations so *forcibly and repeatedly laid down* in the "Surgical Dictionary." *That the trephine must not be applied, nor even an examination made, unless such injuries be actually accompanied by pressing and alarming symptoms of compression.* Inculcations, indeed, rendered doubly influential by the highly respectable sources from which they have emanated, and the channels, also through which they have been so widely disseminated amongst, too, the junior, and, consequently, the less experienced part of the profession.

I must not conclude, however, without observing, that it has not been my object, in this paper, to enter fully into the opinions of Mr. Pott, but rather to point out the dangers which, I conceive, are so liable to result from adopting so opposite an extreme of practice, to that laid down by this very eminent and experienced surgeon.

Nor must I forget to add the no less important than conclusive practical remark of Dr. Thomson, late professor of Military Surgery, in this University. In closing his valuable and highly interesting lectures on "Injuries of the Head." The Doctor says, "I have collected the opinions of many of the most eminent surgeons on the Continent of Europe, respecting the use of the trephine, not one of whom ever had to *regret having performed the operation, whilst they all had, of their not having performed it.*"

---

* Such depressions as the following, will be found, for the most part, perhaps, to do well without the operation.

A farmer's daughter, about eight years of age, fell from a horse. I found a watch-glass-like depression, about an inch in diameter. There was no wound of the integuments. She had vomiting and stupor for some hours, but the next day was nearly well. A strict antiphlogistic regimen was enjoined, and no unpleasant effects ensued. About two years after, I saw the child. The depression had remained.

### II.

*Report of the Physician of the Small-Pox and Vaccination Hospital, February, 3d.* 1825.—Physician, Dr. GEO. GREGORY.*

Of all the diseases which afflict the human race, the most formidable (whether we consider the extent of its ravages, or the intensity of its virulence,) is, unquestionably, the Small Pox. The Plague rages only in particular countries, and at particular seasons, but the Small Pox spreads its devastations over every region of the habitable globe, indifferent alike to summer heat and winter cold. Placed as your Physician is, at the head of the only charitable Institution existing in the world for the express relief of this dreadful disorder, he considers it in some measure his duty, by an annual communication to the Governors, to direct their attention, and that of the public, to a subject so replete with interest; to impress upon them the melancholy, but important truth, that the Small Pox still continues among us, and that incessant attention alone can prevent a renewal of those scenes of death and misery, which rendered the disease an object of such terror to our ancestors.

TABLE OF ADMISSIONS INTO THE SMALL POX HOSPITAL, DURING THE YEAR 1824.

| Class. | CHARACTERS OF THE DISEASE. | Admissions. | Deaths. | Rate of Mortality per Cent. |
|---|---|---|---|---|
| 1 | Small Pox in the Unprotected, in its greatest virulence...... *(Confluent malignant.)* | 12 | 12 | 100 |
| 2 | ——— in its second degree of virulence *(Simple confluent.)* | 46 | 27 | 59 |
| 3 | ——— in its third degree of virulence *(Coherent.)* | 55 | 14 | 23 |
| 4 | ——— in its mild form............... *(Distinct.)* | 35 | 1 | 3 |
| | Total in the Unprotected.. | 148 | 54 | 36 |
| 5 | Small Pox of the Confluent, Coherent, and distinct Kinds, occurring subsequent to Vaccination............... *(Modified.)* | 45 | 0 | 0 |
| 6 | Small Pox occurring subsequent to Inoculation ........... *(Secondary.)* | 2 | 0 | 0 |
| 7 | Eruptive Diseases, not Small Pox ...................... | 4 | 0 | 0 |
| | Total............. | 199 | 54 | 27 |

TABLE OF THE AGES OF THE SEVERAL CASES ADMITTED IN 1824.

| | | | |
|---|---|---|---|
| Under Seven Years of Age ............................ | 33 | 19 | 57 |
| Between Seven and Fourteen .......................... | 15 | 2 | 13 |
| Adults .................................................. | 151 | 33 | 22 |
| Total.............. | 199 | 54 | 27 |
| Total Vaccinated in the year 1824......3,324. | | | |

* We deem the following able Report of Dr. Gregory to be too important for private circulation, and therefore, at our own expence, give it a currency that will at once make its utility general throughout the professional world, on both sides of the Atlantic.

A cursory glance at the proceedings which have taken place within the walls of the Hospital, during the past year, will point out the circumstances which have led your Physician to this train of reflection, and show, at the same time, the extent of benefit which the public has reaped from the labours of your useful Institution.

1. The annexed Tables show, in the first place, that the usefulness of your Hospital, and its credit with the public, are in no degree diminished; for, in the last year, one hundred and ninety-five persons labouring under genuine Small Pox, and four having complaints mistaken in the first instance for Small Pox, have partaken of its benefits; and the annual admissions have never (except in one instance) exceeded the number of two hundred since the commencement of the present century.

2. They point out, in the second place, how great is the mortality of natural Small Pox, when it attacks those who are wholly unprotected. Out of one hundred and forty-eight persons admitted into your Hospital under these circumstances, fifty-four died, being at the rate of thirty-six per cent. They show, further, that children suffer from this disease in a much greater degree than grown persons; that is to say, in the ratio of fifty-seven to twenty-two. This great mortality among children, compared with that which takes place at a more advanced period of life, is well worthy of the notice of the Governors. It arises, doubtless, from the comparative feebleness of the human constitution in infancy, and its consequent incapacity to resist with equal effect the inroads of this severe disorder; but the fact is chiefly deserving of attention, as proving, that any measure calculated to throw the burthen of the disease upon a period of life better able to oppose it (even though it had no pretensions whatever to the character of a *preventive)* would yet confer the most essential benefit on mankind:—and this leads me to state,

3. In the third place, that these Tables are fitted to show, in a manner the most unequivocal, some of the advantages of Vaccination. From them the Governors will perceive, that forty-five cases of Small Pox subsequent to Vaccination have been admitted during the year 1824, all of whom were discharged cured. Yet these exhibited, at their onset, the several degrees of virulence which characterise Small Pox in its purest form, and in the usual proportion; that is to say, some were confluent, some coherent, some distinct and mild. By a comparison of these cases with those in the Table that immediately precede them, it will be apparent, that, without the protecting influence of Vaccination, thirty-six per cent. (that is, sixteen out of the forty-five) would have died. This simple statement appears to your Physician calculated to allay much of that uneasy feeling which the occasional failures of Vaccination excite. It shows, that where Vaccination is incapable of resisting effectually the approaches of Small Pox, it arrests it in its course, and strips it of all its malignity.

Your Physician, however, by no means wishes to rest the merits of Vaccination on this slender and doubtful basis. He is anxious again to impress upon the Governors his firm conviction, strengthened by the experience of another year, that Vaccination is, in a very large proportion of cases, a complete security against the Small Pox; and that the benefits which the Hospital confers upon the public, by the reception of persons labouring under Small Pox, are enhanced an hundred and a thousand fold by the strenuous exertions which it makes to diffuse that inestimable blessing. It is with great satisfaction that your Physician reports, that 3,324 persons have been Vaccinated under his superintendence during the past year, a number exceeding, by 195, that of 1823. The confidence of the public in the vaccine protection is founded, not on the opinion of medical practitioners, but on an extended observation of its utility and safety; and it is highly gratifying, therefore, to find that confidence not only unshaken, but actually on the increase.

Your Physician has availed himself of the opportunities which his office affords of studying minutely the phenomena of Vaccination, and he has directed his inquiries with the view of ascertaining, as far as possible, to what circumstances the occasional failures of Vaccination are owing. He is well convinced, that much yet remains to be discovered in this path of science, but he has seen enough to convince himself, that not a little may be attributed to careless and imperfect Vaccination. It does not seem to have been hitherto well understood, that the human frame is not at all times susceptible of that full and complete beneficial influence which Vaccination is capable of exerting; the consequence of which is, that Vaccination has been often performed in a state of bodily health, which precluded all reasonable hope of deriving benefit from it.

Your Physician is satisfied, that the presence of fever, of diarrhœa, and of inflammation in some internal organ, interferes so materially with the vaccine process, as in many cases to render its protecting powers uncertain, and in some to prevent them altogether. He is convinced that Vaccination has been hitherto viewed too much as a trifling and unimportant operation. Its performance by other hands than those of medical men, is, he believes, nearly, if not altogether, done away with. This affords some prospect of a diminution in the number of failures; but he confidently anticipates still further improvement, when medical practitioners attach a due degree of importance to the operation, and practise it only in such circumstances as afford a fair prospect of obtaining the *full effect* which it is fitted to produce.

Your Physician, in particular, would offer a caution against submitting a child to the process of Vaccination during the period of dentition; and he is further anxious to impress upon those who are engaged in Vaccinating, the necessity of *preparing* the body, in many instances, for its reception; and of keeping the system, during the progress of the disease, free from inflammatory tendency, in the manner formerly practised in regard to Variolous Inoculation. It is quite obvious, that an operation, which is to free the constitution from the influence of a poison so active and subtle as that of Small Pox, should be conducted with, at least, the same degree of attention which was bestowed upon the attempt to introduce it into the system.

Your Physician has thus endeavoured to strike, with a fair and impartial hand, the balance of the defects and benefits of Vaccination. The hope entertained by its illustrious and amiable discoverer, that it might ultimately exterminate Small Pox from off the face of the earth, appears vain and unfounded. The decree of Providence seems to be, that Small Pox shall never cease out of the land. In his mercy he has been graciously pleased greatly to lessen the sphere of its virulence, and to mitigate the intensity of its horrors, but it still exists, and, as far as human eye can penetrate, will for ever continue to exist, one of the many diseases by which mankind is chastised.

With these remarks, your Physician concludes his present Report. The Governors will perceive that he has not shrunk from the investigation of that part of the subject which offered the greatest difficulties, nor hesitated to throw out the suggestions which the limited experience of a few years has dictated to him. He is well aware that these are imperfect. A quarter of a century only has elapsed since the first discovery of Vaccination; and this period is not sufficient to enable us to see fully, and to appreciate justly, the bearings of the many intricate questions connected with Vaccination. He can only assure the Governors, however, of his anxious wish to improve the opportunities which their patronage affords; and, to the utmost of his power, to render the Small Pox Hospital useful to the public at large, as well as to the miserable victims of that dreadful disorder.

GEORGE GREGORY, M. D.

8, Upper John-street, Golden-square,
February 3, 1825.

---

### HOSPITAL FOR THE CASUAL SMALL POX AND FOR VACCINATION, SAINT PANCRAS.

*Committee, February 3, 1825.*

PRESENT,

*FLORANCE THOS. YOUNG, Esq., Chairman,*

*&c. &c. &c.*

RESOLVED,—*That the Thanks of this Committee are justly due to Dr.* GEORGE GREGORY, *for his very skilful, comprehensive, and well-digested Report, now read, and for his assiduous attention to the subjects of Small Pox and Vaccination which it evinces—and which are now most important, not only to the practice of this Hospital, but also to the welfare of the public in general.*

ORDERED,—*That the Secretary be desired to transmit a fair Copy thereof to His Royal Highness the* DUKE OF YORK, *President; and then to cause the same to be printed, and circulated to all the Governors; and afterwards to be distributed at the Hospital.*

[*Extracted from the Minutes.*]

*A. HIGHMORE, Secretary.*

III.

*Sand Rock Spring Cottage,*
*Isle of Wight, February, 7th,* 1825.

Sir,

It having been suggested to me, by some highly respectable medical gentlemen of my acquaintance, that it would be very desirable to obtain the ingredients which enter into the composition of the Aluminous Chalybeate Water, from the Sand Rock Spring in this Island, in a concentrated state; I beg leave to inform you, I have accordingly procured a quantity of the residual Salt, by a slow and careful evaporation of the Water, in a water bath, which from experience, I am enabled to say possesses all the medicinal properties of the Water itself, the chief ingredients of which, being the Sulphates of Iron and Alumine, cannot possibly suffer any decomposition from the low degree of temperature at which the process is conducted.

The dose in which I have usually exhibited this Salt, is about six grains (which is nearly equivalent to an ounce of the Water in its natural state) joined with some Bitter Vegetable Extract, and this I have repeated three or four times a day with the occasional addition of a few grains of the Pill ex Aloe c^m^ Myrrh, as the state of the bowels may require. This form, I have found particularly advantageous in those cases in which the use of the Water itself is either inconvenient or inadmissible. I shall, therefore, feel myself extremely obliged by your giving this a place in your recollection, and when an opportunity offers, that you would prescribe it in such cases as may require the aid of so powerful a tonic.

I further take leave to inform you that in consequence of the very beneficial effects of the Waters, and the great resort of persons to the Spring, several most comfortable Lodging Houses have been erected in its vicinity, for the accommodation of those patients who may prefer taking the Water from its source, and which being situated in the most romantic part of the Undercliff in a climate particularly salubrious, cannot fail to prove of service in those cases in which the administration of the Water is indicated.

I beg to apologise for thus intruding myself on your attention, and
Have the honor to remain, Sir,
Your most obedient humble Servant,

T. L. WATERWORTH.

### IV.

### CROWN LIFE ASSURANCE COMPANY.

CAPITAL £1,500,000,

IN 30,000 SHARES OF £50 EACH.

*Directors.*—WILLIAM PEATT LITT, Esq. Chairman.—JOHN WELLS, Esq. M. P. Deputy-Chairman.—William R. Cosway, Esq.—James Colquhoun, Esq.—James Colvin, Esq.—Captain J.W.D.Dundas,R.N.—James Farquhar, Esq. M. P.—Thomas Harrison, Esq.—George H. Hooper, Esq —John Kirkland, Esq.—Major Moody, *Royal Engineers*.—Sir Francis Ommaney, M. P.—Thomas Solly, Esq.—Alexander Stewart, Esq.—William Whitmore, Jun. Esq.—John Wilson, Esq.—William Wilson, Esq.

*Auditors.*—John Joseph Harrison, Esq.—Isaac Solly, Jun. Esq.—Henry Stock, Esq.

*Trustees.*—John Wells, Esq. M. P.—James Farquhar, Esq. M. P.—Sir Francis Ommaney, M. P.—George Henry Hooper, Esq.—Alexander Stewart, Esq.

*Bankers,*—Messrs. Whitmore, Wells, and Whitmore, Lombard-street.

*Standing Counsel,*—Charles Ellis, Esq.

*Physician,*—Dr. James Johnson, Physician Extraordinary to H.R.H. the Duke of Clarence, Suffolk-place.

*Surgeon,*—James Wardrop, Esq. F.R.S. Ed. and Surgeon Extraordinary to the King, Charles-street, St. James's.

*Solicitor,*—Thomas Haddan, Esq.—*Actuary,*—Mr. J. M. Rainbow.

*Secretary,*—Mr. T. G. Conyers.

The distinguishing feature of this Institution is the extension of the advantages of the system of Life Assurance to classes of individuals who are at present altogether excluded from them, except at premiums quite disproportionate to the actual risk.

It is also intended to combine the ordinary business of Life Assurance upon principles which are the result of the experience of the existing Establishments, and which, whilst they afford adequate security to the Company, will, at the same time, be found to present important advantages to the public.

It is well known that the Officers in His Majesty's Army, Navy and Royal Marines,and in the Military and Maratime Service of the Honourable the East-India Company, are altogether deprived of the benefit of Life Assurance during periods of actual service, except at exorbitant premiums, and that under any circumstances differing from the ordinary risks they experience considerable difficulty in effecting insurances.

The Officers employed in the Merchant Service, a very numerous and respectable class of men, labour under similar disadvantages.

With a view to remove these obstacles, considerable pains have been taken to collect the requisite data for calculating a scale of premiums proportionate to the additional risks arising from actual service, and from sea and climate, and it is satisfactory to state that the lives of gentlemen engaged in the professions before mentioned may be insured at moderate and corresponding rates.

The present Company has therefore been established, having for its objects the ordinary business of Life Insurance, the Insurance of the Lives of Officers in His Majesty's Army, Navy, and Royal Marines, (both at home and *upon actual service,)* in the Military and Maritime Service of the Honourable the East-India Company, and in the Merchant Service of the United Kingdom, and the insurance of the lives of individuals intending to reside abroad, either permanently or for shorter periods, and *of persons proceeding upon single voyages to any quarter of the globe, or remaining at sea,* at premiums proportionate to the actual risk, and calculated upon unquestionable and ascertained data.

This Company will also grant Life Annuities, present and deferred, and Endowments for Children, purchase Life and Reversionary Interests, advance money on Mortgage, or other security, and transact generally all business appertaining to a Life Insurance Company.

Experience having shown the policy of compelling Shareholders to effect Insurances, Shareholders in this Company will be required to insure, or procure and keep on foot Insurances, to the amount of one-half of their Shares, to purchase or sell an Annuity, the consideration for which shall be equal to that amount, or to pay an annual fine of 2*s.* 6*d.* per share upon such Moiety.

Persons assured for the whole term of Life will be entitled to participate in the profits, in the proportion of two-thirds to the Assured, and of one-third to the Proprietors, in such manner and under such regulations as shall be determined by the Deed of Settlement. Parties so entitled to participate will also have an option to have their Premiums reduced, or a bonus added to their Capital, according to the amount of profits.

It is also a fundamental principle of the Company, that no Proprietor is to be answerable for any claims or demands against the Company beyond the amount of his Share in the subscribed Capital, and that the Funds of the Company alone shall be answerable to the Insured.

In carrying the extensive views of this Society into effect it will be the object of the Directors to afford to the different classes of Insurers all those facilities which the peculiar nature of their risks may require; and, upon all occasions, consistently with the security of the Society, to promote the wishes of the different parties insured.

The Deed of Settlement will be prepared under the advice of eminent Counsel, and the Shareholders will be required to sign such a Deed as may be so prepared, and shall receive the sanction of the Directors, with whom alone the regulations and details of the Company are to rest.

The Capital will be raised in 30,000 Shares, of £50 each, and the Shareholders will be required (for the purpose of forming a precautionary fund) to pay a deposit of £5 per share into the hands of the Bankers, at such time as may be appointed by the Directors.

A reserve of Shares has been made for the Officers in His Majesty's Army and Navy, and Royal Marines, and in the East-India Company's and Merchant Service, and for the Outports.

All communications to be addressed to the Chairman of the Company, and left at the Office of Messrs. Gatty, Haddan, Gatty, and Haddan, Angel-court, Throgmorton-street, where Prospectuses may be obtained.

### V.

*Report of the Committee of the Westminster Medical Society, to the Annual General Meeting, February 12th, 1825.*

THE Committee of the Westminster Medical Society, on producing their Annual Report, cannot but congratulate the Members on its present highly flourishing condition. The alterations in the plan of the Society, proposed at the beginning of this session, and which, after full deliberation and discussion, at not less than three special meetings were agreed to by a majority of the Members, have now been in operation for four months, and the Committee beg to call the attention of the Society to the result.

The Committee have the satisfaction of stating, that the attendance of Members on the nights of assembling, has been uniformly larger than in any other session since the foundation of the Society. In the last session particularly (not to mention the previous ones,) it could not have escaped notice, that, although the system of fines for non-attendance was in operation, frequently at the beginning of the evening, not more than five or six Members made their appearance, and they rarely increased beyond eighteen or twenty during the meeting; whereas, since the present plan has been adopted, the number of Members has even exceeded eighty, and has been never less than sixty. This gratifying circumstance the Committee must consider to be partly owing to the greater interest taken in the debates, as now regulated, and partly, perhaps, to the more independent condition of the Society, (as the law to abolish the fines levied on the ordinary Members for non-attendance was passed on November the 27th.)

The admission of new Members has also been more numerous than in any former session. During the last, the number only amounted to twelve; already there have been fifty-two new Members elected in the present session, (although there still remains upwards of two months before its termination) of these, four have been elected Honorary Members: and the Committee remark with pleasure, that the number of practitioners, who have been admitted in the rank of Resident Members, has been much larger than the usual proportion, many of whom have shewn their interest in the welfare of the Society, by taking an active part in the debates.

Since the new regulations respecting the papers and subjects brought forward for discussion, the Committee beg to draw the attention of the Society to the spirited manner with which the debates have been conducted, and to the interest and importance of the questions.

The Committee feel confident that the exertions of the Members will not fall off, but that they will consider, that they are contributing most essentially to the welfare of the Society, by coming forward with proposals to the Committee of subjects for discussion.

The Committee have used their utmost endeavours to find a room for the Meetings of the Society, according to the resolution passed at the General Meeting, October 23rd; but regret that they have hitherto been unsuccessful; as, though they have inspected several, they have been less eligible than the present place of meeting.

The Committee have taken into their consideration the alterations in the Code of Laws, rendered necessary by the improved condition of the Society. These alterations will very shortly be read from the chair; and will be afterwards printed for the use of the Members. The Committee trust also, by the end of the present session to be able to publish a complete list of the Members of the Society.

By the report of the Treasurer, it will be observed, that the finances of the Society are very much improved; and although the receipts, including the fines, last session, were considerably less than the expences, the Committee have the satisfaction to state that, in the present session, the receipts will have so far exceeded the ordinary expenditure, that they believe it will be in their power to add to their funded capital.

Before closing their Report, the Committee would draw the attention of the Members to a proposal for extending the utility, and adding to the permanent respectability of the Society, which has met the decided approbation of all the Members who have considered the subject, including many of its oldest supporters. It is suggested, that when any eligible set of rooms can be procured, they shall be taken for the sole use of the Society; and during the week, shall be opened as reading-rooms for the Members at certain hours of the day: for which purpose, the most useful periodical Medical Journals might be taken in, and a library gradually collected. In furtherance of this project, promises have been already received of donations of books, and of subscriptions to cover the first expences of the necessary Furniture, &c. But as the annual expences will be then very considerably increased, it is further proposed to call a General Meeting on the first Saturday in April, to consider of the propriety of calling upon the Resident and Honorary Members (who constitute the majority of those attending the Meetings, and likely to receive most benefit from the projected change) to pay an annual subscriptlon of one guinea each, in addition to their entrance. And as this regulation cannot well be retrospective, and yet the subscriptions, from the average number of new Members each year, will not amount to a sufficient sum at first, it is hoped that many who are already Members, will voluntarily subscribe, annually, the sum mentioned, by which the Society will at once be enabled to commence the above projected plan.

For this purpose it is recommended that a book be opened, in which those who are favourable to the system, may enter their names,—with the amount, in one list, of their intended donation towards the first expences of the furniture and library—and in another list, as purposing to become annual subscribers. By this method, the Society, upon meeting with suitable rooms, will at once be able to negociate for them, and can then call upon Members for the sums, or portions of the sums, for which they have pledged themselves

Resolutions to this effect will be proposed for the consideration of the present meeting.

*(Signed)* HENRY HUTCHINS, } *Secretaries.*

12*th Feb.* 1825. JOS. WRENTMORE, }

☞ *Meetings every Saturday night, from* 8 *till* 10 *o'Clock, at No.* 51, *Great Marlborough Street.*

---

We are glad to see that Mr. Lizars, surgeon, author of the System of Anatomical Plates, has announced an account of his successful operations for the removal of enlarged ovaria from the female abdomen. In one of these cases, the abdominal cavity, from the sternum to the os pubis, was laid open and an ovarium extracted, which measures 11 inches long by 7½ inches broad, and weighs upwards of 5℔

### VI.

*To the Editor of the Medico Chirurgical Review.*

SIR,—I believe it has been for some time a desideratum to obtain an instrument capable of extracting teeth perpendicularly, and at the same time simple and easy in its construction, and application. I should feel much obliged by your inserting the following description of one which I conceive will answer these intents. I was not aware that any thing of the kind had been before attempted, and was rather surprised to find at the instrument-maker's one, which though very antiquated in its appearance (having been made 50 years ago) was yet somewhat similar in principle, but from the bulk and complexity of its structure, could be of no practical utility.

*Description.* A pair of forceps made tolerably strong and very similar to what are called Cartwright's, but with the handles longer, and the beak turned perpendicularly downwards. A groove extends along the under surface of the instrument between the two points where the blades and handles begin to diverge, in which is to be placed a leg, of about a quarter of an inch in length (capable of sliding backwards and forwards as occasion may require,) with its lower end formed into a small, round nob, which is received into, and nearly enclosed by a cup, thus constituting a complete ball and socket joint. This cup is affixed to a small plate of iron, about the breadth of a nidlar tooth turned down a little at the sides, lined with elastic gum, and forming as it were, a kind of cap for the teeth.

The mode of its application is obvious. The cap is to be placed on the tooth immediately in front of the one intended to be removed ; after taking a firm hold, depress the handle of the instrument, giving it at the same time a little lateral motion, and should there be a vacancy in front of the decayed tooth, the cap must be removed on to the one next in succession.

The above description is probably very unintelligible, but should any one wish to be better informed, he may satisfy himself by calling at Evans and Co's, No. 10, Old Change, St. Paul's. I have only to add that the instrument has been used several times in St. Bartholomew's Hospital, and very satisfactorily so.

Iam, Sir,
Your obedient servant,
JOHN REEVE.

St. Bartholomew's Hospital,
March 10th.

### VII.

*To Dr. James Johnson.*

Dear Sir,

In reply to the enquiries you have made, as to the duty of the Coroner, in regard to opening the bodies of those who have died under suspicious or mysterious circumstances, I have, upon the spur of the occasion, thrown together a few observations which may, perhaps, be of some use, although less digested than, with longer time, I might have been able to present them.

I am not aware that, in point of *law*, it is imperative upon the Coroner to have the body opened. Since my attention has been more particularly directed to medico-legal inquiries, it has been my persuasion that this office stands in much need of reformation; nor am I singular in the opinion that the interests of justice would be promoted, were it confided, generally, to medical men rather than to attornies, as is the prevalent custom. This is the opinion of many judicious individuals, both in the magistracy and of general acquirements, with regard to public economy.

I am not sorry for the opportunity, now afforded me, of adding an observation to what I have said on the burdens of the profession, in my recently published volume on Medical Evidence.* Practitioners are much in the habit of complaining about the great trouble and inconvenience they suffer by frequent attendance on inquests. The Coroner, it is true, can compel personal attendance to give evidence; but I am grievously mistaken if he can require the opening of a body, by virtue of his office. In the majority of cases, the surgeon, upon whom this sort of duty devolves, is employed and paid by the parish; and, in taking the situation of parish surgeon, I look upon it that he enters into a contract to perform such labour as that in question. At the same time I believe that, when so employed, it is customary to grant extra remuneration. When the surgeon is not in that situation, and is required to perform the operation of opening a body, I think he has a right to stipulate for adequate recompense.

Having stated this view of the subject, I would add, that a Coroner can have no right *to prevent* the opening of a body, where, in the opinion of competent judges, (that is, of medical men,) it is necessary. If a medical witness declare that he cannot ascertain the cause of death without having recourse to that process, I do not see how the Jury can discharge their consciences without it; and if that, or any other species of information be necessary for their satisfaction, the Coroner would be most singularly mistaken in his ideas of duty, did he interfere to prevent it.

* Analysis of Medical Evidence, &c. Chapter ii.

The Jury are sworn "diligently to enquire and true presentment make *how, and in what manner*" the deceased came to his death, &c.* A Coroner cannot act but upon view of the body ; and this view must also be taken by the Jury—upon which, and upon the evidence of the witnesses, their verdict is to be formed.

The statute *de officio Coronatoris,* (as old as the year 1275,) prescribes the *visus corporis;* which, although now a mere form, essential to holding the inquest, was certainly ordained, in the first instance, from a notion that it would enable the Coroner's Court to ascertain the cause of death ; for, if we look at some expressions in the statute, we can form no other conclusion than that such was in reality the case.—" It is to be enquired of them that be drowned, or suddenly dead, and after such bodies are to be seen, whether they were so drowned or slain, or strangled by the sign of a cord tied straight about their necks, or about any of their members, or upon any other hurt found upon their bodies, &c." "Also all wounds ought to be viewed, the length, breadth, and deepness, and with what weapons, and in what part of the body the wound or hurt is, &c." The spirit of these passages evidently favours, if it does not indeed enjoin dissection—for how are we to tell whether a body has been drowned, or ascertain the extent of many—of most mortal wounds, without examination of the internal parts ? Mr. Serjeant Hawkins, from whose work, I quote these passages,† says further—" if a Coroner take an inquest after a body hath been so long buried that it may be reasonably presumed that the view of it could be of no manner of use to the Jurors, &c." All this is singularly precise if we look at the practice even of medical men down to a recent period : for they themselves were accustomed to set great importance on the external aspect of the body in such cases, and the now indispensible duty of examining the internal parts is almost of late introduction.

But the Jury is sworn to decide, also, upon the evidence of the witnesses ; and these again are to declare the truth, the whole truth, and nothing but the truth. Now, when a medical man is summoned before the Coroner, it is in the capacity of a witness, not in any official connexion with the Court. He comes under this obligation ; and, let me ask, what medical man, of common probity and ordinary professional acquirements, could pronounce upon the cause of death without discovering it ? How can he declare the whole truth if not acquainted with the whole of the matter that imparts that truth ? How can he be clear in his own mind that he may not say something which is *not the truth,* if not persuaded as to the nature of the circumstances that constitute the truth of the matter ?

The Coroner is certainly bound to encourage, and (if he cannot order) to procure the opening of bodies in all cases requiring medical evidence. If a *superficial* view of a body will do, any Juryman may know as much of the matter as a medical man ; but if the aid of the latter be

* Burn's Justice. † Pleas of the Crown.

necessary, it must be in order to instruct the Court; and the Court cannot direct him in what way they are to be instructed. It is a rule with regard to evidence, that the best is to be procured;* and the Coroner is bound to receive all the evidence produced by either party. Now it may so happen that, if the one party chuses to forego the process of dissection, the other may insist upon it—and the course for the Coroner to pursue in such a case is laid down.

It was not long since ruled in the Court of King's Bench,† that the Coroner is to direct the Jury in point of law, and to explain to them the distinction between murder and manslaughter. Of course it must be his duty to ascertain whether there be grounds for an accusation of either; and if the medical witnesses are not permitted to seek for evidence of violence, an unlawful impediment is thrown in the way of justice; and the Coroner is liable to heavy penalties, as well as loss of office for neglect of his duty. On this point, I shall take the liberty of referring to the case of the woman who died from the bursting of an abscess in the liver, during an altercation with her husband.‡

Juries have fully as little acquaintance with the medical, as they have with the legal, bearings of matters that come before them; and are often disposed to express themselves satisfied, while yet totally uninformed about them. It would sound strange even to the Coroner, did they dispense with his information about the *law* of their duty, but he often acquiesces in their statement of satisfaction with the *science* of it. If his object be not merely to earn his fees, he will not refer the merits of the case to their decision until these merits have been laid before them in the fullest manner possible. How, otherwise, can their opinion be sworn to as a *vere dictum?* Much in all cases will depend on the Coroner's discretion, and on the intelligence of Juries—but there is much also dependent on the medical witness. If medical men were not, in general, so inept for the discharge of their forensic duties, we should see a very different course pursued on many inquests.

Such statements as the foregoing do not require much legal investigation, and are, perhaps, not less entitled to the attention of the medical profession, that they are not communicated by a lawyer. The sources whence they are derived are open to every one, and familiar to many who have no connexion with juridical pursuits. The inferences I have ventured to draw must be obvious to every one, in the habit of reasoning.

I remain, yours truly,
J. G. SMITH.

March 12, 1825.

---

* Phillips on the Law of Evidence, ch. vii. sect. 6.

† By Mr. Justice Bailey, on the application for a mandamus, that arose out of the notorious Oldham Inquest, in 1819.

‡ Principles of Forensic Medicine. The case has been repeatedly referred to by subsequent writers as an important illustration.

